# Orthodontic Management of Skeletal Class II Malocclusion with the Invisalign Mandibular Advancement Feature Appliance: A Case Report and Review of the Literature

**DOI:** 10.1155/2022/7095467

**Published:** 2022-05-13

**Authors:** Melina Koukou, George Damanakis, Apostolos I. Tsolakis

**Affiliations:** ^1^School of Dentistry, Department of Orthodontics, National and Kapodistrian University of Athens, Thivon 2, Goudi, 11527 Athens, Greece; ^2^Orthodontic Department, School of Dental Medicine, Case Western Reserve University, Cleveland, Ohio, USA

## Abstract

The treatment of Class II malocclusion due to mandibular retrognathia is one of the most common challenges met in orthodontic practice. When it comes to a growing patient, functional appliances are the optimal way to achieve growth modification by enhancing mandibular growth. Clear aligners have been part of the orthodontic treatment for several decades but until recently they were only used to correct mild malocclusions. In 2017, Align Technology introduced the Invisalign with Mandibular Advancement Feature (IMAF) which replicates the action of functional appliances. As this device is new to clinical practice, there is limited literature on its clinical efficiency. This case report describes the orthodontic management of a 12-year-old male patient having skeletal Class II malocclusion due to mandibular retrognathia. As the patient was experiencing active growth, the IMAF appliance was chosen for his treatment. The IMAF appliance appears to be successful in the treatment of Class II malocclusion with mandibular retrognathism in a growing patient. As with all functional appliances, the correction of the malocclusion is a result of both skeletal and dental effects and the IMAF presents the advantage of producing less proclination of the lower incisors compared to other functional appliances.

## 1. Introduction

The idea of using a tooth positioning appliance for the alignment of teeth was first introduced by Kesling in the 1940s [1]. At the time, Kesling's positioner could only produce minor tooth movements through tipping of the crowns and was mainly used at the end of the orthodontic treatment for the final result refinement [2]. The philosophy behind the clear aligner is the manufacturing of an appliance made on a model of the teeth in the desired position. The evolution of this idea was the manufacturing of a sequence of aligners on a series of tooth models to progressively reach the ideal position of the teeth [2, 3]. Sheridan et al. further evolved this type of treatment by combining it with interproximal reduction (IPR) [3–5]. Clear aligners were originally manufactured on wax up casts from impressions of the teeth, which made the treatment very time-consuming and therefore inefficient [2, 3]. Advancements in dental materials and computer technology in the last decades allowed for a much easier and efficient use of clear aligners and such appliances begun gaining popularity [6–9]. In 1997, Align Technology introduced the Invisalign appliance, the first system to incorporate CAD-CAM technology [2, 3, 10, 11].

At present, there is a wide variety of appliances included in the term clear aligner and they are distinguished into (a) conventional and (b) 3D designed and printed aligners [2, 9]. Conventional aligners are not a result of 3D technology and without any auxiliary accessories achieve tooth movement only by the aligner itself; these represent aligners for minor tooth movements that patients can even use without regular dental appointments [2, 9]. Examples of such systems are MTM clear aligner, Originator, Simpli 5, Clearguide System, Crystal Braces, Smile Care, and Suresmile. 3D designed and printed aligners are more sophisticated appliances that have incorporated CAD-CAM technology and bonded resin attachments which enable for a larger scale of tooth movements [2, 7, 9]. The most common of them are Invisalign, ClearCorrect, ClearPath, eCligner, K Line, and Orthocaps [7, 9]. All these systems in order to achieve a more complex treatment use tools including ellipsoid, beveled and rectangular attachments, pressure points, bite ramps, power ridges, and several features of classic orthodontics such as intermaxillary elastics, interproximal reduction, and power arms [2, 7, 9].

There is a great debate in the literature about which cases can be successfully treated with clear aligners. Initially, only mild malocclusions could be addressed, but as the systems evolve and incorporate more elements in the treatment, the complexity of malocclusions treated with clear aligners constantly increases. Attempts have been made to define exactly what movements are possible with clear aligners but more research is needed in the field for a definite conclusion on the contemporary techniques regarding clear aligners. A review of the literature suggests that horizontal movements can be more accurately produced but posterior rotations and movements in the vertical dimension such as intrusion or extrusion of the teeth and occlusal contacts appear to be more difficult [5–7, 9, 12–15].

Class II malocclusion is one of the most prevalent orthodontic problems, with mandibular retrognathism presenting in most of the cases [16–19]. For Class II treatment, a variety of appliances have been used over the years in order to enhance mandibular growth by posturing the mandible in a forward position [16, 20]. The ideal time for this treatment is during the growth spurt of the patient as maximum growth modification may occur [19, 20]. Functional appliances are the choice for this treatment and they are distinguished in fixed functional appliances such as the Herbst, the Powerscope, and MARA and removable functional appliances such as the Twin Block, the Bionator, Frankel, and activators [16, 19, 20]. The most common use of the fixed appliances is the Herbst and of the removable appliances the Twin Block [19, 21, 22].

The question of how much of the results seen by functional appliances is due to skeletal or dental effects or even to the natural course of growth has been the subject of extensive research. D'Antò et al. and Cacciatore G et al. found some evidence supporting increase in mandibular length and improvement of the SNB angle with functional appliances but reported that there is still no sufficient evidence to support clinical significance of the appliances [23, 24]. In a systematic review by Cozza et al., it is reported that in two-thirds of the included studies, clinically significant supplementary mandibular elongation was achieved, with Herbst appliance and Twin Block being the most efficient devices [16]. The Herbst and the Twin Block appliances have also been reported as the most effective functional appliances by several studies [18, 21, 23]. Santamaría-Villegas et al. found significant increase in mandibular length concluding in the effectiveness of functional appliances in mandibular advancement, in agreement with previous studies [18, 20, 25, 26].

The correction of the Class II malocclusion occurs as a result of a combination of skeletal and dentoalveolar effects and it is generally believed that fixed appliances have more dental effects than removable appliances [17, 20, 27–29].

In 2017, Align Technology introduced the Invisalign with Mandibular Advancement Feature (IMAF). This device replicates the action of functional appliances as it features buccal “precision wings” usually between the 1st molar and premolars which can only interlock when the patient postures the mandible forward (a mechanism similar to Twin Block) while simultaneously correcting malocclusion and crowding issues [19, 30].

As this appliance is new to clinical practice, the aim of this study is to present the treatment of skeletal Class II malocclusion with the IMAF. This case report describes the orthodontic management of a 12-year-old male patient having skeletal Class II malocclusion due to mandibular retrognathia. As the patient was experiencing active growth, the IMAF was chosen for his treatment.

## 2. Case Presentation

### 2.1. Diagnosis and Etiology

A 12-year-old male patient presented for orthodontic consultation. His main concern was the excess anterior overjet which he perceived as prominent teeth. He had no relevant family history, no significant prenatal, post-natal, and medical history, and no history of parafunctional habits.

**Figure 1:**
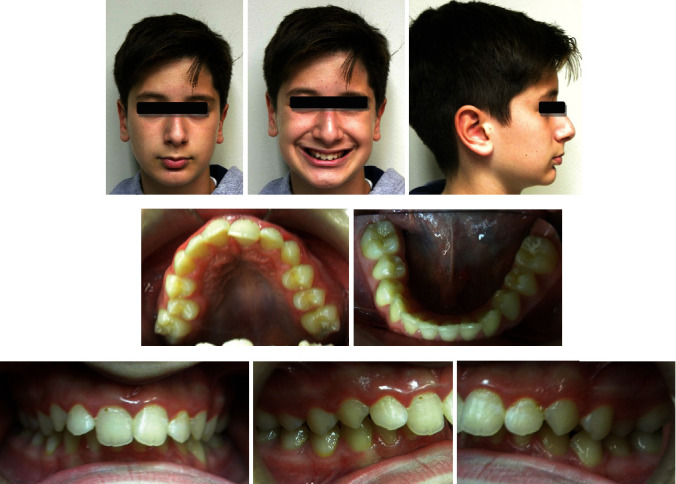
Pretreatment photographs.

The clinical examination revealed a convex facial profile, short anterior lower face height, and midline deviation of the maxilla, the mandible, and the chin to the right (Figure 1).

**Figure 2:**
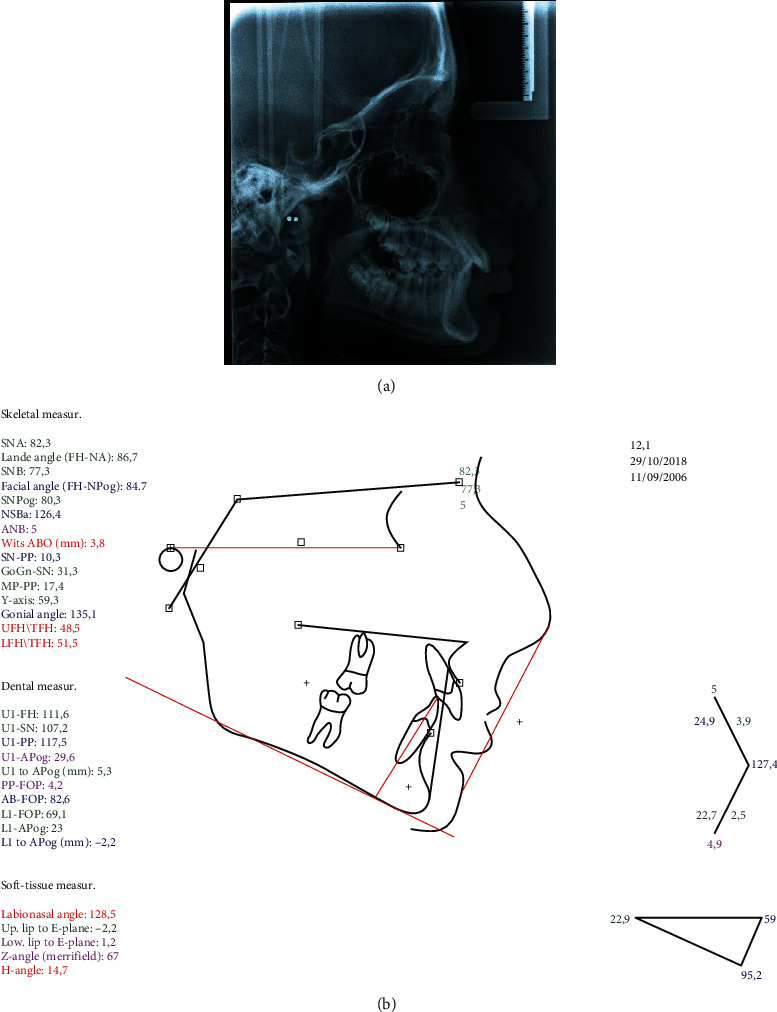
Pretreatment (a) lateral cephalogram and (b) tracing.

**Figure 3:**
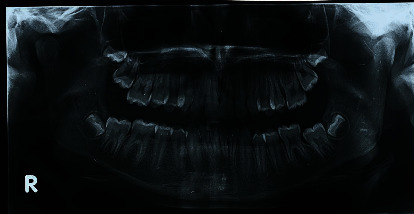
Pretreatment orthopantomogram.

On intraoral and radiographic examination, he had permanent dentition, upper arch crowding, and a Class II, division 1 malocclusion with a deep bite (Figures 1–3).

The cephalometric analysis showed a skeletal Class II antero-posterior discrepancy with an ANB angle of 5° (SNA: 82.3°, SNB: 77.3°) due to mandibular retrognathia (FH-NPog: 84.7°) (Figure 2).

### 2.2. Growth Evaluation

The patient was a 12-year-old male and therefore was in active growth stage. According to Baccetti et al.'s stages of cervical vertebral maturation, he was between cervical stage 2 (CS2) and 3 (CS3) and the peak of his growth spurt was expected in a year from the moment of the initial evaluation. [31]

### 2.3. Treatment Objectives

The treatment objectives were to correct upper spacing and deep overbite, establish Class I molar and canine relationship, achieve optimal occlusion, maintain facial balance, and improve dental and facial esthetics.

### 2.4. Treatment Plan

Since the patient had a skeletal Class II pattern and as he was in active growth stage, growth modification with the Invisalign Mandibular Advancement Feature appliance was planned.

### 2.5. Treatment Stages

The first phase of the treatment included a set of 16 clear aligners (aligners #01-16) in order to align and level the dental arches and expand the upper dental arch. The aligners were changed weekly for 4 months.

After the first four months, the mandibular advancement (MA) phase was followed with two “bite jump” mandibular advancements of 3.3 mm increments each. The aligners were changed weekly and each “bite jump” was achieved by 14 aligners (aligners #17 to #30 for the first 3.3 mm bite jump with a duration of 14 weeks; aligners #31 to #44 for an additional 3.3 mm bite jump for 14 weeks). Consequently, the mandibular advancement phase lasted 7 months.

A stabilization phase followed for 1 month with a set of 3 aligners (aligners #45-47) which had precision wings but were not programmed to produce any further mandibular advancement.

**Figure 4:**
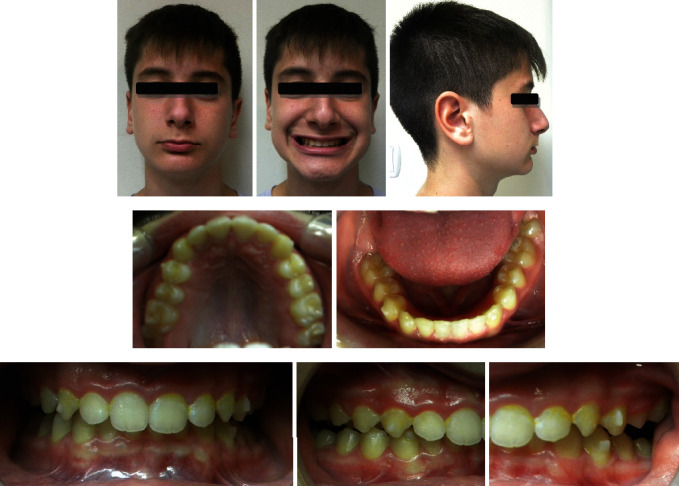
Photographs after the MA phase.

**Figure 5:**
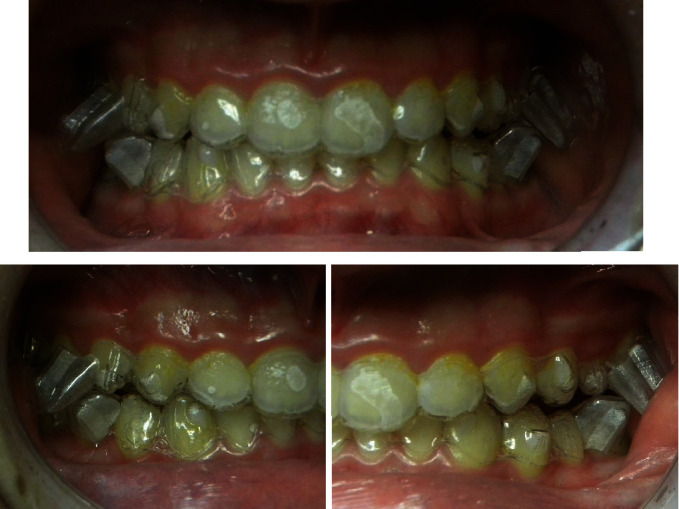
Photographs with IMAF in place.

**Figure 6:**
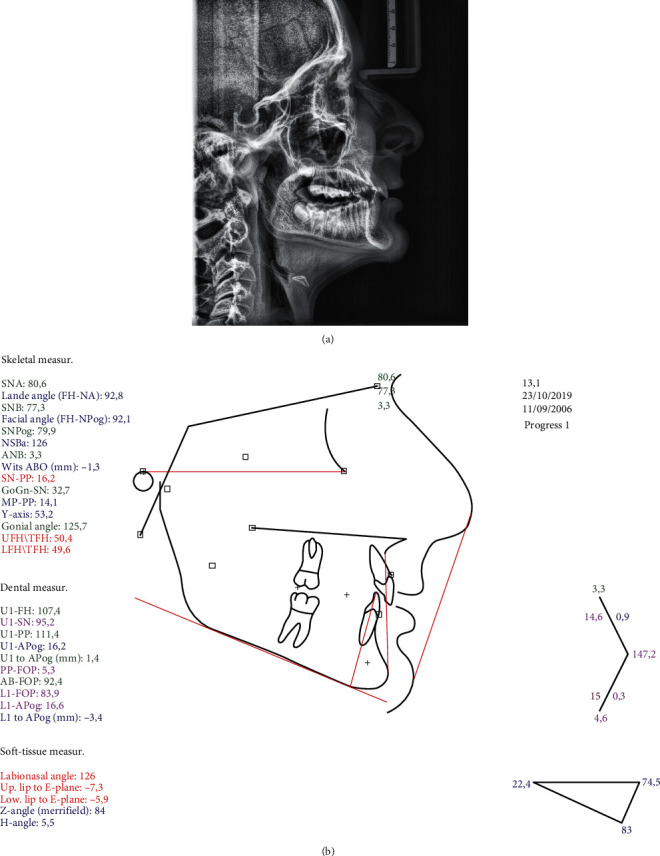
(a) Lateral cephalogram and (b) tracing after MA phase.

**Figure 7:**
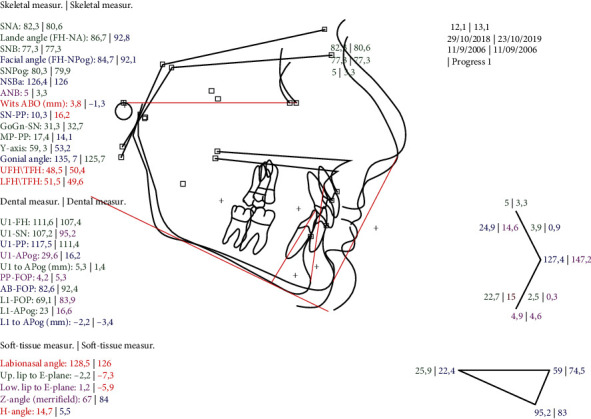
Superimposition on the FH plane pretreatment and after the MA phase.

After the MA phase, the Class II relationship was corrected, the facial profile was improved, and the mandible was advanced. A posterior open bite appeared in the area of the teeth beneath the precision wings (Figures 4–7).

Subsequently, the patient was re-scanned and a set of 26 aligners in combination with Class II elastics were used in a 7-month period for detailing and correcting the posterior open bite that occurred from the use of precision wings in the MA phase.

### 2.6. Treatment Results

**Figure 8:**
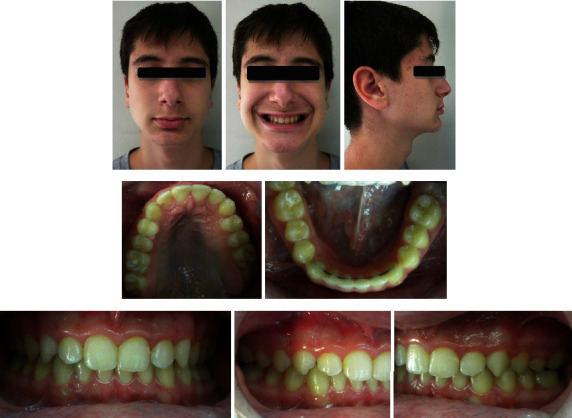
Post-treatment photographs.

**Figure 9:**
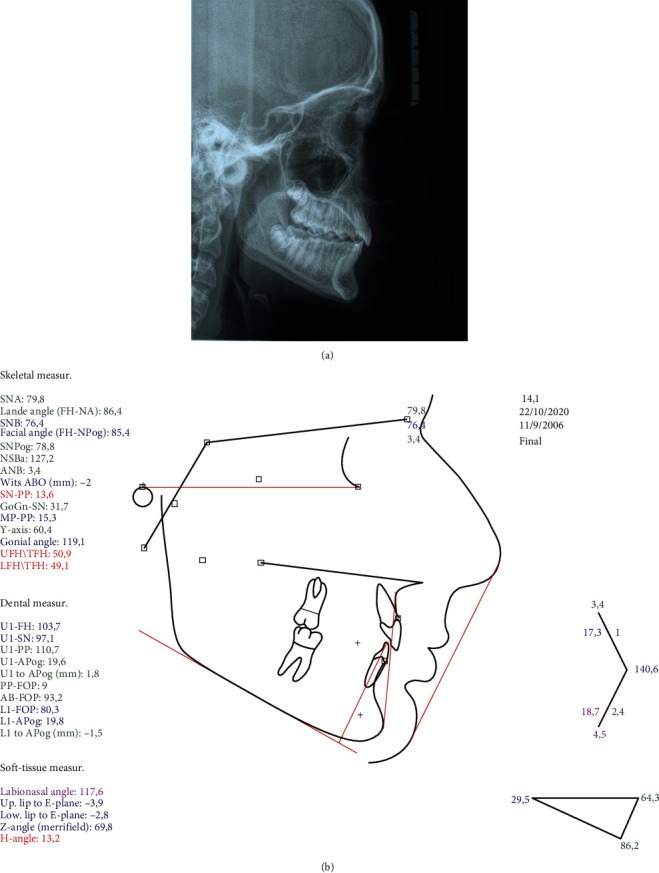
Post-treatment (a) lateral cephalogram and (b) tracing.

**Figure 10:**
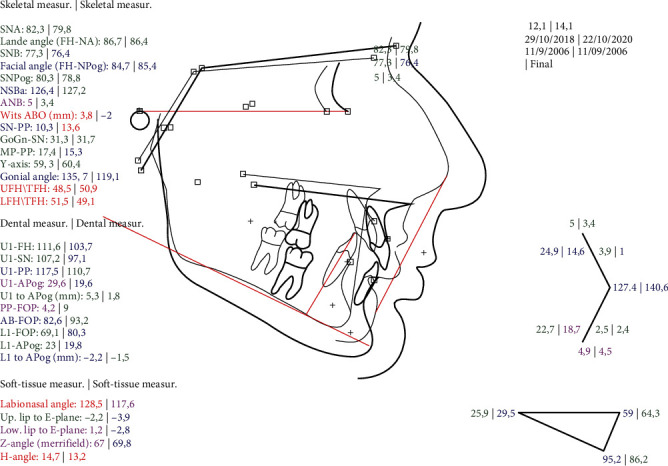
Superimposition on the FH plane pretreatment and post-treatment.

At the end of the treatment, there was a significant improvement in the patient's profile and facial esthetics. The overjet and overbite were corrected with a Class I canine relationship and a super Class I molar relationship. The dental and facial midlines were in alignment (Figures 8–10, Table 1).

## 3. Discussion

As the IMAF appliance is new to clinical practice, there is limited literature on its efficiency, consisting mostly of case studies. Blackham conducted a retrospective cephalometric study to measure the skeletal, dental, and soft tissue effects of the IMAF. The results of this study indicate that the IMAF is effective in improving skeletal and soft tissue convexity, the Wits appraisal, and the ANB angle [19]. These results are in agreement with a retrospective controlled study by Caruso et al. and a prospective controlled study by Ravera et al. who also found the IMAF to be effective in improving face convexity [32] and the Wits index [33]. Blackham found that the overjet was decreased through retraction of the upper incisors and protrusion of the lower incisors and the overbite was also reduced [19]. Ravera et al. showed that if the patients were at CVM2 growth stage, the IMAF appliance would produce more dentoalveolar effects whereas if the patients were at CVM3 growth stage, the skeletal component of the Class II correction was greater. [33] As with all functional appliances therefore, correction of the Class II malocclusion with the IMAF appliance is achieved through both skeletal and dental changes [19, 32, 33].

Blackham's study shows that the IMAF is very similar to Twin Block, with some differences in the timing that the growth modification is completed with each device and with less proclination of the lower incisors with the IMAF when compared to the Twin Block [19]. The good control of incisor proclination is also supported by Caruso et al. and Ravera et al. [32, 33].

Regarding the vertical plane, Caruso et al. reported that the IMAF produced no significant changes in FH-PP and MP-PP [32] and Blackham also reported no significant changes in SN-MP, FMA, and P-A Face Height [19]. Mandibular length was increased in a lower level when the initial length of the mandible was longer or when the patients were older [19].

The patient of this care report with mandibular retrognathism was successfully treated with the IMAF appliance one year before the peak of his growth spurt. The standard treatment with IMAF consists of two “bite jump” mandibular advancements of 2 mm, each achieved by 8 aligners. In this case, we preferred to proceed with two “bite jump” mandibular advancements of 3.3 mm increments each. The aligners were changed weekly and each “bite jump” was achieved by 14 aligners. This gradual mandibular advancement appears to be more effective than the classic approach of a single jump [33].

As expected, after treatment, the ANB angle, the Wits index, and the overall face convexity of the patient were improved. Part of the correction of the Class II malocclusion was achieved through dental compensation of the upper arch, but on the lower arch, the lower incisors were not proclined any further after treatment, as often happens with functional appliances; in fact, they were slightly retroclined.

## 4. Conclusions

The IMAF appliance appears to be successful in the treatment of Class II malocclusion with mandibular retrognathism in a growing patient. As with all functional appliances, the correction of the malocclusion is a result of both skeletal and dental effects and the IMAF presents the advantage of producing less proclination of the lower incisors compared to other functional appliances.

## Figures and Tables

**Table 1:** Comparative cephalometric parameters.

Cephalometric parameters	Pretreatment values	Values after MA phase	Post-treatment values
SNA (°)	82.3	80.6	79.8
SNB (°)	77.3	77.3	76.4
ANB (°)	5	3.3	3.4
Facial angle (FH-NPog) (°)	84.7	92.1	85.4
Wits ABO (mm)	3.8	-1.3	-2
MP-PP (°)	17.4	14.1	15.3
LFH/TFH (°)	51.5	49.6	49.1
U1-SN (°)	107.2	95.2	97.1
L1-MP (°)	95.2	83	86.2
Interincisalangle (°)	127.4	147.2	140.6

## Data Availability

The data used to support the findings of this study are included within the article.
